# Regulated in Development and DNA Damage Responses -1 (REDD1) Protein Contributes to Insulin Signaling Pathway in Adipocytes

**DOI:** 10.1371/journal.pone.0052154

**Published:** 2012-12-18

**Authors:** Claire Regazzetti, Karine Dumas, Yannick Le Marchand-Brustel, Pascal Peraldi, Jean-François Tanti, Sophie Giorgetti-Peraldi

**Affiliations:** 1 INSERM U 1065, Mediterranean Research Centre for Molecular Medicine, Team: Cellular and Molecular Physiopathology of obesity and diabetes, Nice, France; 2 University of Nice Sophia Antipolis, UFR Medicine, Nice, France; 3 UMR CNRS 7277, UMR INSERM 1091, Faculty of Medicine, Nice, France; CRCHUM-Montreal Diabetes Research Center, Canada

## Abstract

REDD1 (Regulated in development and DNA damage response 1) is a hypoxia and stress response gene and is a negative regulator of mTORC1. Since mTORC1 is involved in the negative feedback loop of insulin signaling, we have studied the role of REDD1 on insulin signaling pathway and its regulation by insulin. In human and murine adipocytes, insulin transiently stimulates REDD1 expression through a MEK dependent pathway. In HEK-293 cells, expression of a constitutive active form of MEK stabilizes REDD1 and protects REDD1 from proteasomal degradation mediated by CUL4A-DDB1 ubiquitin ligase complex. In 3T3-L1 adipocytes, silencing of REDD1 with siRNA induces an increase of mTORC1 activity as well as an inhibition of insulin signaling pathway and lipogenesis. Rapamycin, a mTORC1 inhibitor, restores the insulin signaling after downregulation of REDD1 expression. This observation suggests that REDD1 positively regulates insulin signaling through the inhibition of mTORC1 activity. In conclusion, our results demonstrate that insulin increases REDD1 expression, and that REDD1 participates in the biological response to insulin.

## Introduction

REDD1 (Regulated in development and DNA damage response 1) also known as DDIT4 or RTP801 has been identified as a stress-induced protein in 2002 [1], [2]. REDD1 is a 25 kDa ubiquist protein with a low level of expression in basal conditions. Its expression is induced in response to hypoxia, stress and DNA damages through the activation of distinct transcription factors. Hypoxia and CoCl_2_ stimulate REDD1 expression through HIF-1 (Hypoxia Inducible Factor) transcription factor [2], [3], while oxidative stress and reticulum endoplasmic stress regulate REDD1 expression through ATF4-C/EBPβ and DNA damages by p53/p63 [1], [4], [5].

REDD1 acts as an inhibitor of mTORC1 (mammalian Target Of Rapamycin Complex-1) activity. mTOR integrates several extrinsic signals that regulate cell growth and metabolism. mTOR is the catalytic component of two multiproteins complexes, mTORC1 and mTORC2. Even if the two complexes are composed of mTOR, they are activated through different mechanisms and display different cellular functions. mTORC1 regulates the rate of protein synthesis by controlling mRNA translation initiation and progression, ribosome biogenesis and autophagy, while mTORC2 regulates actin cytoskeletal organization and cell polarization [6], [7].

In response to growth factors, mTORC1 is activated through the inhibition of TSC2. Growth factors activate PKB (protein kinase B), which phosphorylates TSC2 (Tuberous Sclerosis 2). This phosphorylation induces the association of TSC2 with 14-3-3 proteins and the inhibition of its GAP activity towards Rheb, allowing the activation of mTORC1 by GTP-Rheb protein.

REDD1 is an inhibitor of mTORC1 in several cellular models. It has been proposed that REDD1 functions through the regulation of the TSC1/TSC2 complex. Indeed, in response to hypoxia, REDD1 would sequester 14-3-3 proteins apart from TSC2 [8], leading to the activation of TSC2 GAP activity and the inhibition of mTORC1. However, the precise mechanisms remain to be elucidated since the structural analysis of REDD1 failed to substantiate this observation [9].

Post-translational regulation of REDD1 has been studied, and it has been demonstrated that REDD1 protein has a short half life and is regulated through protein degradation mechanisms depending on GSK3 (Glycogen Synthase Kinase 3) phosphorylation [1], [10], [11]. This suggests that post translational regulation of REDD1 could play an important role in the regulation of its level of expression.

In obesity and diabetes, modulation of mTORC1/S6K-1 has been involved in the development of insulin resistance. Insulin resistance is a ubiquitous correlate of obesity and a central component of type 2 diabetes and is characterized by a decreased response to insulin, linked to perturbation of insulin signaling. Activation of insulin receptor leads to phosphorylation of substrates such as IRS and Shc triggering PI-3-kinase/PKB and Ras-ERK pathways [12]. Several mechanisms are involved in the development of insulin resistance: decrease in insulin receptor kinase activity and recruitment of negative adaptors proteins such as SOCS3 or Grb7/10/14 [13]–[15]. Another mechanism of insulin resistance involves the serine phosphorylation of IRS-1 [16], [17]. This serine phosphorylation is mediated by various serine/threonine kinases, among them mTORC1/S6K. Thus, chronic activation of mTORC1/S6K induces insulin resistance through a negative feedback loop involving the serine phosphorylation and the degradation of IRS-1.

Interestingly, while it activates mTORC1, insulin is also a known inducer of REDD1 transcription. This has been observed in skeletal muscle [18] and in adipocytes where we have demonstrated that the mechanism is dependent upon HIF-1 [19].

Since mTORC1 participates in insulin signaling pathway, we have explored the role of REDD1 in insulin signaling. In particular, we have addressed (i) whether REDD1 stability is regulated by insulin and (ii) the function of REDD1 in insulin pathway.

In the present study, we demonstrated that short term treatment with insulin increases REDD1 expression through a MEK-dependent pathway. Moreover, we show that REDD1 is required for an adequate insulin signaling pathway in adipocytes through a mechanism dependent of mTORC1. Together, these results demonstrate that REDD1 is an important partner of insulin pathway that plays the role of a biological rheostat.

## Materials and Methods

### Materials

Insulin was obtained from Lilly (Paris, France). Antibodies were obtained from the following companies: REDD1 - Proteintech (Chicago, IL); phosphotyrosine, phospho-S235/236 S6, phospho-Thr389 S6 kinase, phospho-Thr421/Ser424 S6 kinase, phospho-Thr308 PKB, phosphor-Ser473 PKB, phospho-Thr202/Tyr204 ERK1/2, ERK1/2, MEK- Cell Signaling Technology (Beverly, MA); ERK2, HSP90 and IR - Santa Cruz Biotechnology (Heidelberg, Germany); tubulin - Sigma-Aldrich; V5– Invitrogen; Nedd4 and IRS-1 - BD Biosciences. Control siRNA and two different siRNA directed against REDD1 (#1:s93077 and #2:s93078) were purchased from Ambion (Foster City, CA) and ON-TARGETplus SMARTpool mouse REDD1 siRNA (#3:74747) was obtained from Thermo Scientific. The primer sets for real time PCR were purchased from Eurogentec (Seraing, Belgium). Culture media were obtained from Invitrogen (Carlsbad, CA). Inhibitors were obtained from Calbiochem (Nottingham, UK).

### Plasmids

#### pCEP4- CA MEK

A constitutively active form of MEK (CA-MEK) was obtained by deleting the region encompassing amino acids 32–51 and by mutating the two serine residues (218 and 222) to aspartic acid. This form of MEK possesses a high level of kinase activity [20]. **pCMV-REDD1.** The REDD1cDNA was generated by reverse transcription using mRNA from hypoxic DU145 cells and subcloned into Flag-pCMV expression vector (Sigma). All mutants were generated by the QuikChange site-directed mutagenesis kit (Stratagene). Sequences of the constructs were checked by sequence analysis.

CUL4-V5 and DDB1-V5 expression vectors were obtained from Pr. Raychaudhuri (University of Illinois, Chicago) and Dr. Helen Piwnica-Worms (Howard Hughes Medical Institute, Washington University, St Louis). Plasmid encoding for Nedd4 has been described [21].

### Cell Culture and Transfection

3T3-L1 fibroblasts were obtained from ATCC (CL-173), and grown and induced to differentiate in adipocytes as previously described [22]. Briefly, 3 days after confluence, 3T3-L1 fibroblasts were treated for 2 days with DMEM–10% FCS (vol/vol) supplemented with isobutyl methylxanthine (250 nmol/l), dexamethasone (250 nmol/l), rosiglitazone (10 µmol/l) and insulin (800 nmol/l), and then for two additional days with DMEM–10% FCS containing 800 nmol/l insulin. Adipocytes were used between days 2 and 7 after the end of the differentiation protocol when the adipocyte phenotype appeared in more than 90% of the cells. The isolation and properties of hMADS cells have been described in Plaisant et al. [23]. Human embryonic kidney cells (HEK-293) were maintained in Dulbecco’s modified Eagle’s medium (DMEM) containing 10% (v/v) fetal calf serum.

Transient transfection of HEK-293 cells was performed by Fugene (2 µg of DNA/9.4 cm^2^) according to manufacturer instructions.

#### Transfection of siRNA

3T3-L1 adipocytes were used for reverse transfection 7 days after induction of differentiation. 3T3-L1 adipocytes were trypsinized, control siRNA, or siRNA directed against REDD1 (40 pmol) were transfected using INTERFERin (Polyplus Transfection) according the protocol of Kilroy and al [24]. Briefly, siRNA complexes (80 nM final concentration) were incubated with INTERFERin and lay onto the wells. 3T3-L1 adipocytes were trypsinized and added to the siRNA/INTERFERin complexes solution. The adipocyte phenotype after transfection was assessed by visualization of lipid droplets, staining with oil red O and expression of PPARγ protein (unpublished data).

### Western Blot Analysis

Serum-starved cells were treated with ligands, chilled to 4°C, and washed with ice-cold phosphate-buffered saline (6 mmol/l Na_2_HPO_4_, 1 mmol/l KH_2_PO_4_, pH 7.4, 140 mmol/l NaCl, 3 mmol/l KCl) and solubilized with RIPA buffer (50 mmol/l Tris pH7.5, 150 mmol/l NaCl, 1% NP40, 0.1% SDS, 0.5% Na Deoxycholate, 1 mmol/l Orthovanadate, 5 mmol/l NaF, 2.5 mmol/l Na_4_P_2_O_7_ and Complete protease inhibitor cocktail (Roche Diagnostics, Meylan, France) for 30 min at 4°C. Lysates were centrifuged (14,000 rpm) for 10 min at 4°C, and the protein concentration was determined using BCA protein assay reagent (Thermo Fisher Scientific, Brebières, France). Cell lysates were analyzed by Western blot. Immunoblots were revealed using a Fujifilm LAS-3000 imaging system. Quantifications were realized using MultiGauge or ImageJ softwares.

### mRNA Analysis

RNA was isolated from adipocytes (TRIZOL, Invitrogen), and cDNA was synthesised using Transcriptor first strand cDNA synthesis kit (Roche Diagnostics, Meylan, France). Relative quantification of gene expression was measured by real-time PCR. The primer sets used for REDD1 were (F) 5′-TCCCTGGACAGCAGCAACA-3′ and (R) 5′-AACGACACCCCATCCAGGTA-3′ and for the house keeping gene 36B4 (F) 5′-TCCAGGCTTTGGGCATCA-3′ and (R) 5′-CTTTATCAGCTGCACATCACTCA-3′.

### Lipogenesis

3T3-L1 adipocytes were incubated with [^3^H] sodium acetate (0.4 mmol/l, 0.5 µCi/ml) in absence or in presence of insulin 1 nM for 1 hour at 37°C. Cellular lipids were extracted with a mixture of Butyl-PBD-Toluene and the radioactivity in the organic phase was determined in a liquid scintillation counter.

#### Statistical analysis

Statistical differences between groups were analyzed by Student’s *t* test and were considered significant at *P* < 0.05.

## Results

### Insulin Induces REDD1 Expression through a MEK Dependent Pathway

**Figure 1:**
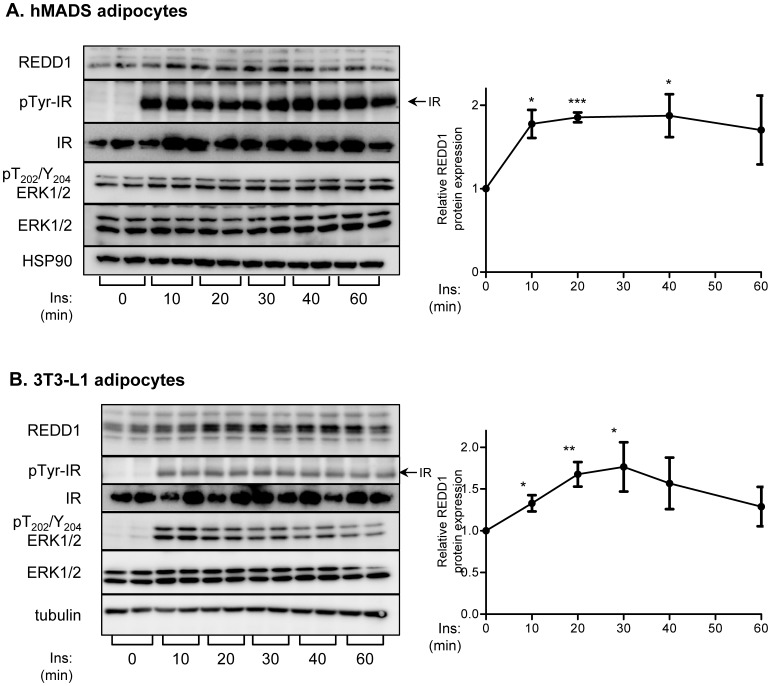
Insulin stimulates REDD1 expression in human and murine adipocytes. hMADS adipocytes (A) and 3T3-L1 adipocytes (B) were stimulated with insulin (100 nM) for the indicated period of times and analyzed by immunoblots with indicated antibodies. Quantification of three independent experiments done in duplicate is shown and REDD1 protein level is expressed compared to HSP90 or tubulin expression (*p<0.05; **p<0.01, ***p<0.001).

**Figure 2:**
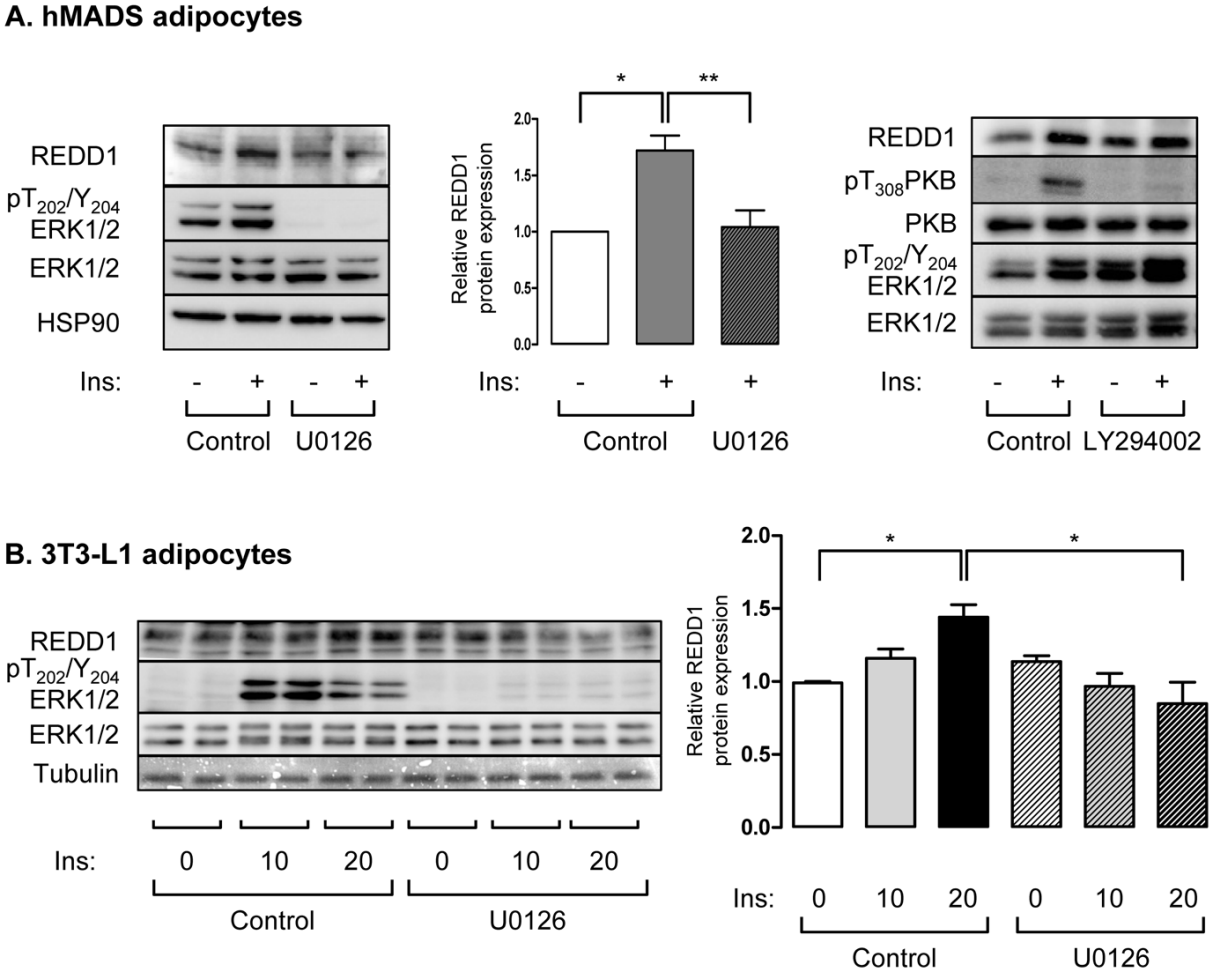
Insulin stimulates REDD1 expression through MEK dependent pathway in hMADS adipocytes. (A) hMADS adipocytes were stimulated with insulin (100 nM) for 20 minutes in absence or in presence of a pretreatment with U0126 (10 µM) or LY 294002 (50 µM) for 60 minutes. (B) 3T3-L1 adipocytes were stimulated with insulin (100 nM) for 10 and 20 minutes in absence or in presence of a pretreatment with U0126 (10 µM). Proteins were revealed by immunoblots using indicated antibodies. REDD1 protein level is expressed compared to HSP90 or tubulin expression and quantification of three independent experiments is shown (*p<0.05; **p<0.01).

Since REDD1 has a half life of 5 min [10], we have investigated whether insulin could modulate its stability. We have used two models of cultured adipocytes, human mesenchymal stem cells differentiated in adipocytes (hMADS) and murine adipocytes (3T3-L1). Fully differentiated hMADS and 3T3-L1 adipocytes were stimulated with insulin from 0 to 60 minutes, and REDD1 protein levels and phosphorylation of ERK1 and ERK2 were evaluated by Western blots (Figure 1). In both cell types, insulin stimulated the phosphorylation of its receptor and the phosphorylation of ERK. Short-term insulin treatment stimulated REDD1 protein expression. After 20 minutes, insulin induced a 1.9 fold increase in REDD1 protein level. After 60 minutes, REDD1 levels remained elevated in hMADS adipocytes while its expression was transient in 3T3L1 adipocytes (Figure 1B). In order to identify the signaling pathway implicated in the regulation of REDD1 expression in response to insulin, we used U0126, a potent and specific pharmacological inhibitor of MEK1/2, the upstream kinase of ERK1/2 [25]. In Figure 2, hMADS adipocytes were treated with U0126 or with LY294002, a PI-3-kinase inhibitor, prior being stimulated with insulin. We observed that U0126 inhibited insulin-induced REDD1 expression, whereas LY294002 treatment had no significant effect. These results suggest that insulin-induced REDD1 expression is mediated through a MEK/ERK dependent pathway. In 3T3-L1 adipocytes, inhibition of ERK pathway also inhibited insulin-induced REDD1 expression (Figure 2B).

### Constitutive Active MEK Increases REDD1 Protein Expression

**Figure 3:**
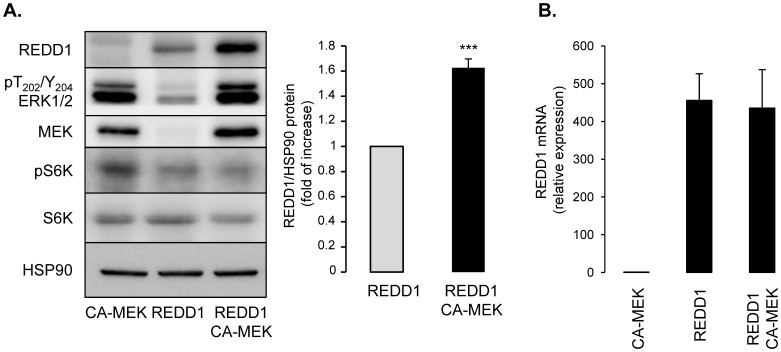
Expression of constitutive active MEK increases REDD1 protein level in HEK-293 epithelial cells. HEK-293 cells were transfected with plasmids encoding for constitutive form of MEK (CA-MEK), REDD1 or both plasmids. (A) Proteins were analyzed by immunoblots using indicated antibodies. REDD1 protein level is expressed compared to HSP90 expression and quantification of seven independent experiments is shown (***p<0.001). (B) REDD1 mRNA level was determined by quantitative RT-PCR.

To study the role of MEK in REDD1 expression, we have overexpressed a constitutive form of MEK. Since cDNA plasmid transient transfections in 3T3-L1 adipocytes give a low rate of efficiency, transfections were performed in HEK-293 cells. HEK-293 cells were transfected with expression vectors encoding for REDD1-Flag, a constitutive active form of MEK (CA-MEK) or both. REDD1 and MEK protein levels and phosphorylation of ERK, and S6K (Thr 389) were evaluated by Western blots. As expected, expression of a CA-MEK enhanced ERK phosphorylation (Figure 3A). Interestingly, co-transfection experiments showed that CA-MEK expression led to an increased protein level of the transfected REDD1. Expression of REDD1 is accompanied by a decrease in phosphorylation of Thr389-S6K, which reflect the ability of REDD1 to inhibit mTOR signaling pathway [26]. Co-expression of REDD1 and CA-MEK did not aggravate the inhibition of S6K phosphorylation, probably because the maximum of mTORC1 inhibition mediated by REDD1 has been reached. As a control, CA-MEK did not modify mRNA levels of transfected REDD1 (Figure 3B). These results indicate that expression of a constitutive form of MEK increased REDD1 protein level independently of an increase in its transcription.

### Constitutive Active MEK Protects REDD1 from Proteasomal Degradation

**Figure 4:**
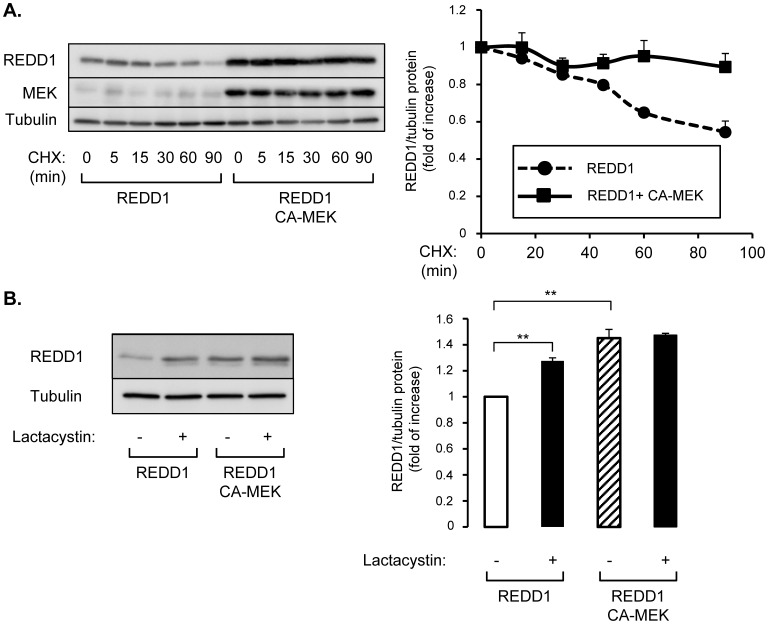
Expression of constitutive active MEK inhibits REDD1 degradation. (A) HEK-293 cells were transfected with plasmids encoding for REDD1 in absence or in presence of a plasmid encoding for constitutive form of MEK (CA-MEK). 48 h after transfection, wells were treated with cycloheximide (CHX 10 µg/ml) for the indicated periods of time, and proteins were analyzed by immunoblots with indicated antibodies. REDD1 protein level is expressed compared to tubulin expression and quantification of three independent experiments is shown. (B) HEK-293 cells were transfected with plasmids encoding for REDD1 in absence or in presence of a plasmid encoding for a constitutive form of MEK (CA-MEK). 48 h after transfection, cells were treated with lactacystin (10 µM) for 5 hours. Proteins were analyzed by immunoblots with indicated antibodies. Quantification of three independent experiments is shown and REDD1 protein level is expressed compared to tubulin expression (**p<0.01).

Since expression of CA-MEK increased REDD1 protein level, we have investigated whether MEK could stabilize REDD1. HEK-293 cells were transfected with REDD1 in absence or in presence of CA-MEK, and treated with cycloheximide (CHX) for up to 90 min (Figure 4A). As observed, in presence of CHX, REDD1 protein half life was about 90 min. In presence of a CA-MEK, REDD1 expression was not statistically modified after 90 minutes of cycloheximide treatment. These results indicate that MEK protects REDD1 from degradation.

The role of MEK in the regulation of REDD1 degradation by the proteasome was assessed using lactacystin, a proteasome inhibitor. HEK-293 expressing REDD1 alone or in combination with CA-MEK were treated with lactacystin (Figure 4B). Lactacystin increased REDD1 protein level suggesting that REDD1 is degraded through proteasome. On the other hand, the increase in REDD1 protein induced by CA-MEK was unaffected by lactacystin. These observations suggest that MEK protects REDD1 from proteasomal degradation.

**Figure 5:**
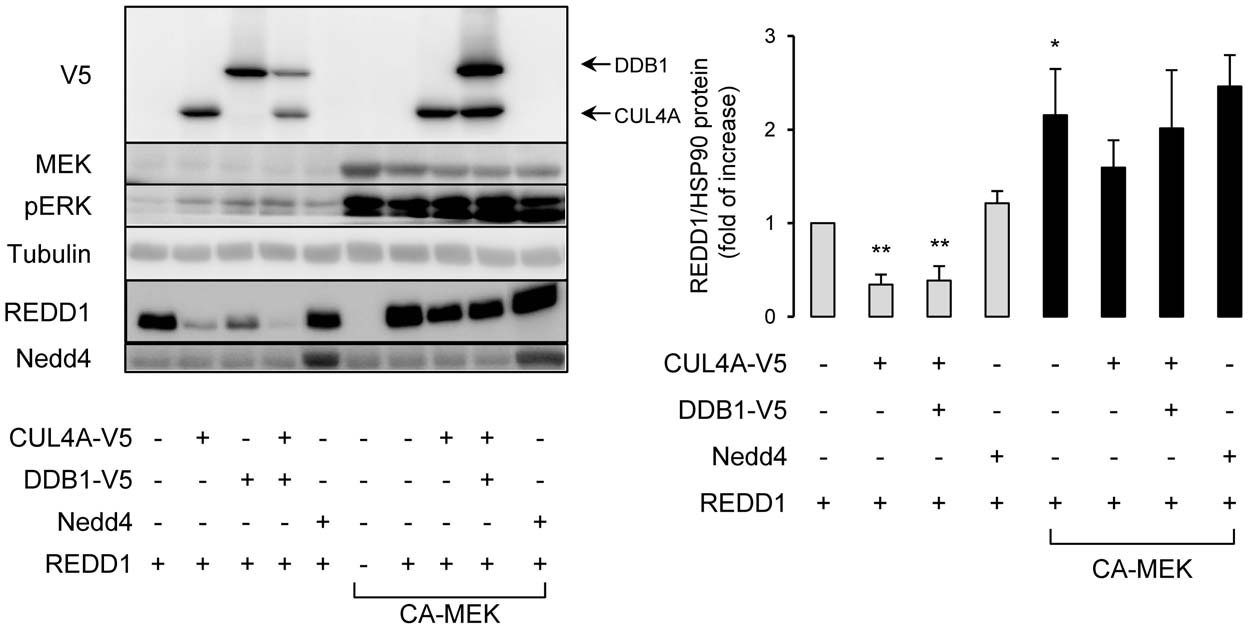
Constitutive active MEK inhibits CUL4A-induced REDD1 degradation. HEK-293 cells were transfected with indicated plasmids. 48 h after transfection, proteins were analyzed by immunoblots using indicated antibodies. REDD1 protein level is expressed compared to HSP90 expression and quantification of three independent experiments is shown (**p<0.01).

Proteasomal degradation of a protein requires its ubiquitination. Ubiquitination is a three-steps process in which ubiquitin is activated by an ubiquitin-activating enzyme (E1), transferred to an ubiquitin-conjugating enzyme (E2) and finally linked to the substrate by an E3 ubiquitin ligase. E3 ubiquitin ligases are mainly separated in two major families, RING and HECT E3 ubiquitin ligases. CUL4A (Cullin 4A), a prototype of RING E3 ubiquitin ligases, has been proposed to mediate ubiquitination and degradation of REDD1 [11]. We have investigated whether MEK could regulate the degradation of REDD1 induced by CUL4A. We have transfected REDD1 in presence of CUL4A, alone or in combination with DDB1 (DNA Damage Binding protein-1), which acts as a linker to recruit substrates, or in presence of Nedd4, a HECT E3 ubiquitin ligase (Figure 5). Expression of CUL4A, DDB1 or both, but not Nedd4, induced a 66% decrease in REDD1 protein level. This degradation was inhibited by CA-MEK. This suggests that REDD1 is degraded through its ubiquitination mediated by CUL4A E3 ubiquitin ligase by a process antagonized by CA-MEK.

### REDD1 has a Positive Action on Insulin Signaling Pathway

**Figure 6:**
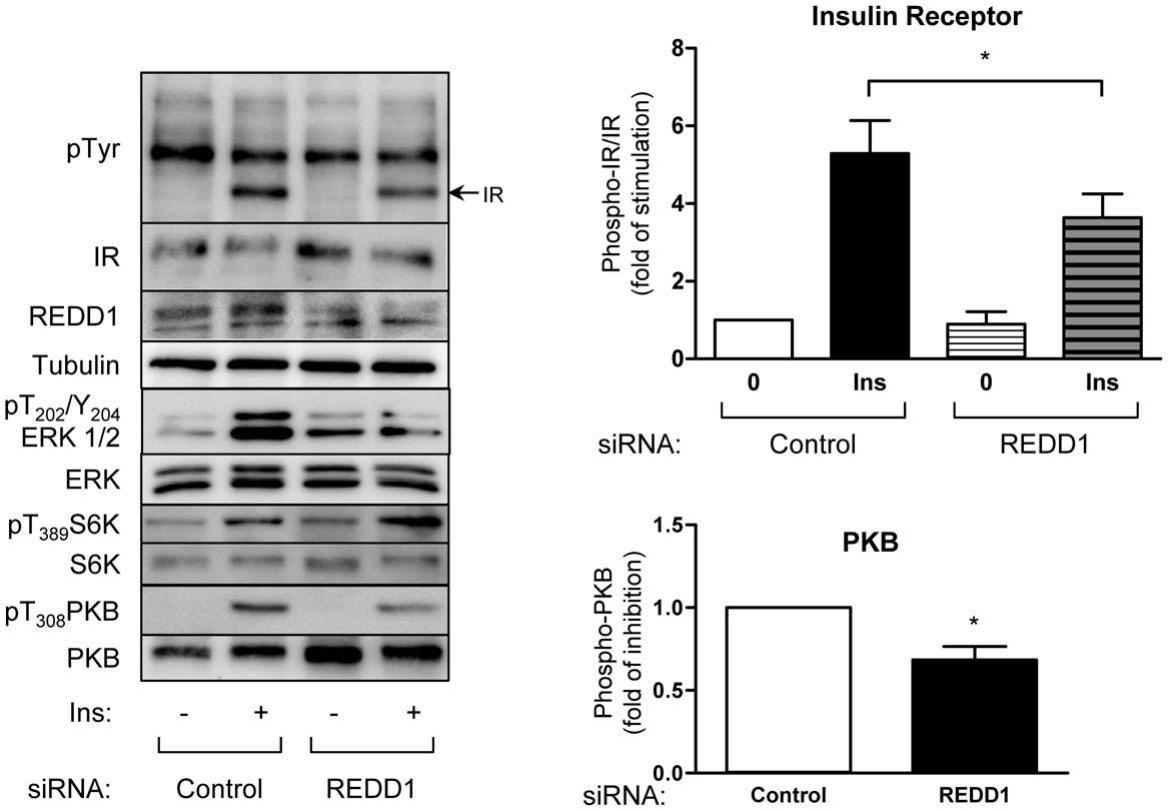
Downregulation of REDD1 expression inhibits insulin signaling pathway in 3T3-L1 adipocytes. 3T3-L1 adipocytes were transfected with REDD1 siRNA 48 h after transfection, adipocytes were stimulated with insulin (10 nM) for 5 minutes. Proteins were analyzed by immunoblots using indicated antibodies. Quantification of phosphorylation of insulin receptor (n = 6) and PKB (n = 5) in response to insulin is shown (*p<0.05).

Next, we studied the role of REDD1 on insulin signaling pathway using siRNA against REDD1. 3T3-L1 adipocytes were transfected with siRNA and treated with insulin (Figure 6). siRNA induced a 55% (+/−3%) decrease in REDD1 protein expression. Because REDD1 is an inhibitor of mTORC1, the decrease in REDD1 expression was associated with an increase of the phosphorylation of S6 kinase, a substrate of mTORC1. Unexpectedly, the decrease in REDD1 expression was associated with a decrease in insulin-induced phosphorylation of insulin receptor (IR) as well as ERK and PKB phosphorylation. This observation was confirmed using three distinct siRNA (Figure S2).

We also monitored the phosphorylation of S6K on Thr421/Ser424 located in the auto-inhibitory domain. Phosphorylation of Thr421/Ser424-S6K is due to ERK activity [27]. We observe that silencing of REDD1 leads to a decrease in ERK activity followed by the inhibition of phosphorylation of Thr421/Ser424 S6K (Figure S3).

**Figure 7:**
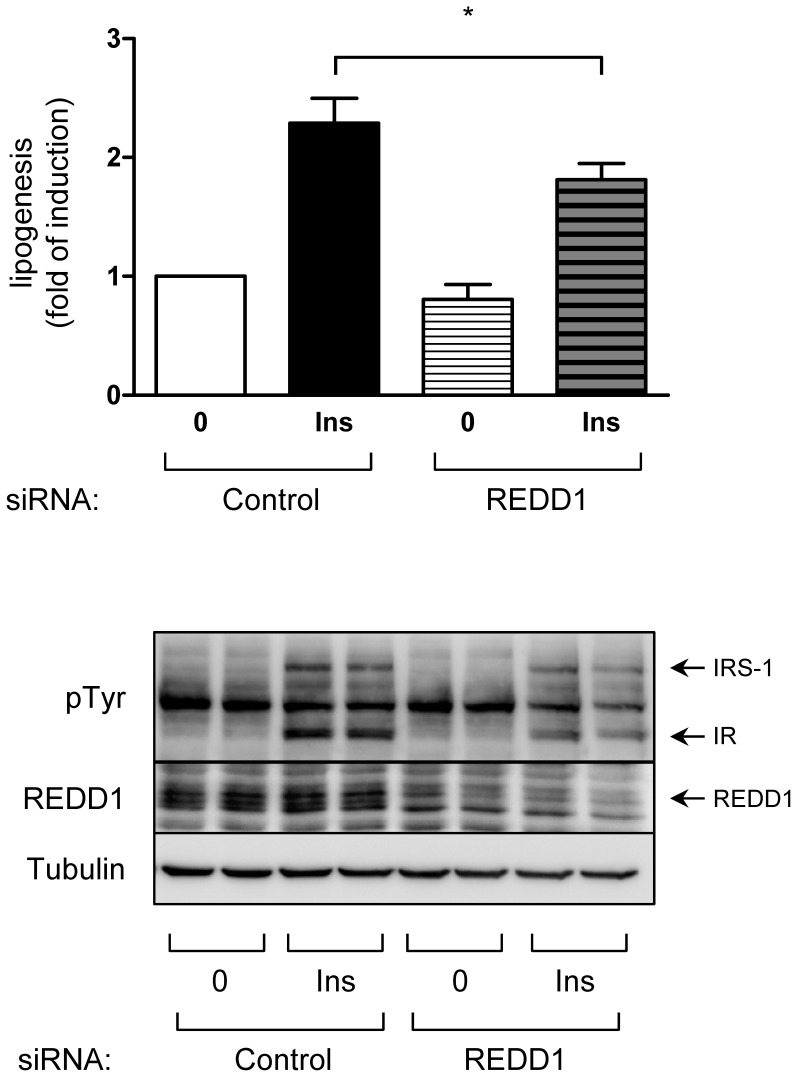
Downregulation of REDD1 expression inhibits lipogenesis in 3T3-L1 adipocytes. 3T3-L1 adipocytes were transfected with REDD1 siRNA. 48 h after transfection, 3T3-L1 adipocytes were incubated with [^3^H] sodium acetate (0.4 mmol/l, 0.5 µCi/ml) in absence or in presence of insulin 1 nM for 1 hour at 37°C. Radioactivity incorporated in cellular lipids was determined in a liquid scintillation counter. Results are expressed as fold of induction of pmol of acetate/mg of proteins (n = 3, *p<0.05). Proteins were analyzed by immunoblots using indicated antibodies.

To evaluate whether this effect could be linked to a biological response, we invalidated REDD1 using siRNA in 3T3-L1 adipocytes and evaluate IR tyrosine phosphorylation and lipogenesis (Figure 7). REDD1 siRNA decreased IR tyrosine phosphorylation. This effect was accompanied with a net decrease in insulin-induced lipogenesis indicating that the inhibition of IR signaling by REDD1 inhibition has biological consequences.

Since mTORC1 activity inhibits insulin signaling [17], we tested whether the inhibition of insulin signaling observed after REDD1 silencing is due to mTORC1 activation.

**Figure 8:**
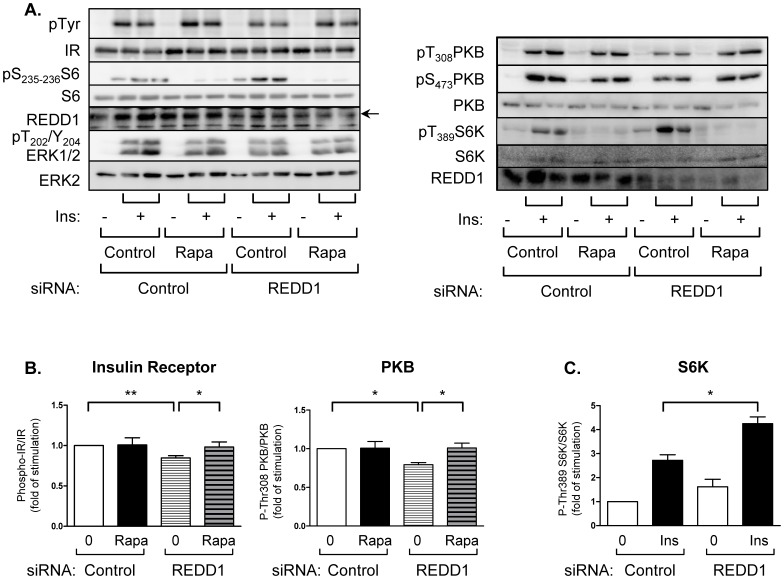
Inhibition of insulin receptor phosphorylation is dependent of mTOR activity. (A) 3T3-L1 adipocytes were transfected with REDD1 siRNA. 48 h after transfection, adipocytes were pretreated with rapamycin (40 nM) for 1 hour before being stimulated with insulin for 5 minutes. Proteins were analyzed by immunoblots using indicated antibodies. (B) Quantification of phosphorylation of insulin receptor (n = 8), pThr308-PKB (n = 3) in insulin-induced conditions is shown. (C) Quantification of phosphorylation of pThr389-S6K (n = 3) is shown (*p<0.05, **p<0.01).

To this end, we have inhibited mTORC1 using rapamycin. In Figure 8, 3T3-L1 adipocytes were transfected with REDD1 siRNA and stimulated with insulin in absence or in presence of rapamycin. Silencing of REDD1 increased of the phosphorylation of S6 kinase and its substrate S6, which reflects the activation of mTOR, and decreased insulin-induced phosphorylation of insulin receptor and PKB (Figure 8). Treatment with rapamycin restored the ability of insulin to stimulate the phosphorylation of its receptor, as well as the phosphorylation of PKB.

In conclusion, our results show that mTORC1 activity is involved in the decrease of insulin signaling pathway observed after downregulation of REDD1. This observation suggests that REDD1 could be required for a proper insulin signaling pathway.

## Discussion

Positive and negative feedback loops are classical elements of signaling pathways that have been selected through evolution to achieve homeostasis of biological processes. All these processes are necessary to finely tune the intensity, the kinetic, the stability and the specificity of the final signal [28]. Mammalian target of rapamycin complex 1 (mTORC1) appears at the crossroad of several signaling pathways and is a classical example of a protein involved in a negative feedback loop. Indeed, mTORC1 is activated by insulin and is necessary for the metabolic and mitogenic responses of the cell to this hormone. However, mTORC1 is also involved in the lowering of the insulin signaling through a negative feedback loop that is highly effective, since it targets the very first steps of insulin signaling [17].

Here, we describe a novel level of complexity of these feedback loops since we show that, in addition to stimulating mTORC1, insulin controls the level of REDD1, an inhibitor of mTORC1.

First, we show that short treatment with insulin controls REDD1 expression. Insulin promotes a rapid and transient REDD1 augmentation in a MEK/ERK dependent pathway. Indeed a specific inhibitor of MEK obliterates the effect of insulin on REDD1 stability in adipocytes and ectopic expression of constitutive active MEK mimics insulin effect on REDD1 expression. Insulin stimulates REDD1 expression through an increase of its transcription in adipocytes and in skeletal muscle [18], [19], and we propose that insulin rapidly (20–30 minutes) stimulates REDD1 expression through the inhibition of its degradation. McGhee et al., have failed to detect any effect of insulin on REDD1 expression in muscle [29]. This discrepancy compared to our results is due to the fact they used a different time course of insulin action and that they did not analyze the effect of insulin in normal rat, only in diabetic refed animals.

AMPK did not seem to be implicated in insulin-induced REDD1 expression in adipocytes, since insulin did not significantly stimulate AMPK (Figure S1). Regulation of REDD1 expression by AMPK is controversial, since AMPK is not required for REDD1 function in response to energy stress [30], whereas in head and neck squamous cell carcinomas REDD1 expression is associated with the activation of AMPK in response to hypoxia [31].

Through a mechanism that remains to be resolved, MEK/ERK pathway seems to control REDD1 degradation. Indeed, expression of CA-MEK prevents degradation of REDD1 induced by cycloheximide or by expression of E3 ubiquitin ligase complex CUL4-DDB1. It can be noted that in our experiments, REDD1 half-life is prolonged compared to what have been observed by Kimball and colleagues, probably because we monitored the degradation of transfected REDD1 protein, whereas Kimball et al. measured the degradation of endogenous REDD1 protein in MEF [10]. Katiyar al al. have demonstrated that REDD1 is degraded after its phosphorylation by GSK3 and its ubiquitination by CUL4A-DDB1 ubiquitin ligase [11]. The molecular mechanisms involved in the stabilization of REDD1 by MEK are still unknown, but we did not observed any modification of GSK3 activation by expression of CA-MEK in HEK-293 cells (unpublished data). A recent study has shown that CUL4A activates ERK pathway, and that U0126 inhibits CUL4A-induced invasion in prostate cancer cells [32]. Even if our results suggest a different regulation of CUL4A through an ERK dependent mechanism, one can envision that both pathways are inter regulated.

Then, we show that REDD1 participates to the insulin signaling pathway. Indeed, silencing of REDD1 in adipocytes inhibits insulin receptor tyrosine phosphorylation and downstream proteins, such as PKB and ERK. Phosphorylation of PKB on Thr308 and Ser473 is decreased, indicating that the kinases responsible for these phosphorylations, PDK1 and mTORC2 respectively, are inhibited. mTORC2 is activated independently of TSC1/TSC2 [33]. Since REDD1 inhibits TSC1/TSC2 activity, silencing of REDD1 will not modulate mTORC2, but only mTORC1 activity.

ERK activation by insulin is also inhibited after downregulation of REDD1. Since the two sites, Thr421 and Ser424 of S6K, are direct substrates of ERK [27], the decrease of ERK activity is accompanied with a decreased in the phosphorylation of these sites.

Moreover, the inhibition of insulin signaling leads to the inhibition of metabolic responses such as lipogenesis. mTORC1 regulates lipogenesis, glucose transport, glycolysis and the expression of genes involved in lipid biosynthesis [33]. The question whether REDD1 modulation of expression could regulate all these metabolic events remains open. As REDD1, DEPTOR, is a protein that represses mTOR signaling. Silencing of DEPTOR expression in 3T3-L1 adipocytes blocked PKB activation and inhibits glucose uptake and incorporation into lipids [34].

The downregulation of insulin signaling observed after inhibition of REDD1 expression is dependent upon mTORC-1, since rapamycin, an inhibitor of mTOR, reverses the inhibitory effect of REDD1 depletion.

The precise mechanism by which REDD1 controls the inhibitory effect of mTORC-1 on insulin signaling remains to be identified. Indeed, mTOR activates a negative feedback mechanism involving serine phosphorylation and degradation of IRS-1. We have been unable to detect any significant modulation of IRS-1 serine phosphorylation or stability (unpublished data), suggesting the existence of another mechanisms controlled by REDD1 and through mTORC-1 inhibition. Since the very first step of insulin signaling, i.e. insulin receptor tyrosine phosphorylation, is affected, it is likely that the target is the insulin receptor itself. For instance, TNF-α an inducer of insulin resistance have been shown to directly affect the insulin receptor [35]. Grb10, mTORC-1 substrate, has also been demonstrated to inhibit insulin signaling [36], [37]. However, downregulation of Grb10 expression with specific siRNA did not reverse the effect of silencing REDD1 expression, suggesting that Grb10 might not be implicated in the inhibition of insulin receptor phosphorylation after suppression of REDD1 expression (unpublished data).

By its action as an inhibitor of mTORC1 activity, REDD1 plays a role in tumor suppression, and genetic ablation of REDD1 induces tumor formation [8]. REDD1 has also a role in muscle atrophy, since its expression is increased in response to chronic hypoxia and its overexpression decreases muscle fiber size [38]. Moreover, its expression is increased in muscle of streptozotocin-induced diabetic mice [39]. Food deprivation induces elevation of circulating glucocorticoids, and is associated with a decreased of mTORC1 activity. It has been demonstrated that food deprivation, through the elevation of corticosterone, stimulates REDD1 expression and that REDD1 is a direct target of glucocorticoid receptor [29], [40].

The role of REDD1 in adipose tissue metabolism remains to be studied. However, our results demonstrate that REDD1 is involved in insulin signaling pathway in adipocytes. We propose that insulin through the activation of MEK/ERK pathway inhibits REDD1 protein degradation. This transient increase of REDD1 protein level is necessary for the insulin signaling pathway. Long-term insulin treatment stimulates REDD1 expression through a PI-3-kinase/mTOR dependent pathway and the activation of HIF-1 transcription factor [19]. Since hyperinsulinemia and hypoxia, which induce insulin resistance [22], lead to a more pronounced and stable increase of REDD1 expression, this suggest that a deregulation of REDD1 expression could be implicated in insulin resistance development.

Together our data unraveled a novel level of complexity in insulin signaling. Indeed, we show here that, by controlling REDD1 stability through MAP kinase pathway, insulin modulates the negative feedback loop induced through mTORC-1 activation. Through regulation of REDD1, insulin tightened its control on mTORC-1 activity. This could allow insulin to control and diversify the intensity and kinetic of activation of the various proteins involved in its signaling pathway. A total understanding of all the molecules involved in the positive and negative feedback loop of insulin signaling as well as their cellular localization and the intensity of their action will be necessary to have a complete picture of insulin signaling.

## Supporting Information

Figure S1 **hMADS adipocytes (A) and 3T3-L1 adipocytes (B) were stimulated with insulin (100 nM) for the indicated period of times and analyzed by immunoblots with indicated antibodies.**
(PDF)

Figure S2 **3T3-L1 adipocytes were transfected with REDD1 siRNA (#1: s93077, #2: s93078, #3: 74747) as mentioned in materials and methods. 48 h after transfection, adipocytes were stimulated with insulin (10 nM) for 5 minutes.** Proteins were analyzed by immunoblots using indicated antibodies.
(PDF)

Figure S3 **3T3-L1 adipocytes were transfected with REDD1 siRNA. Adipocytes were stimulated with insulin for 5 minutes.** Proteins were analyzed by immunoblots using indicated antibodies.
(PDF)
