# Supplementary material for: Regulated in Development and DNA Damage Responses -1 (REDD1) Protein Contributes to Insulin Signaling Pathway in Adipocytes
Source: PLoS One. 2012 Dec 18;7(12):e52154. doi: 10.1371/journal.pone.0052154 (PMC3525563; doi:10.1371/journal.pone.0052154)
Supplement: Figure S1 — hMADS adipocytes (A) and 3T3-L1 adipocytes (B) were stimulated with insulin (100 nM) for the indicated period of times and analyzed by immunoblots with indicated antibodies. (PDF) [file pone.0052154.s001.pdf]

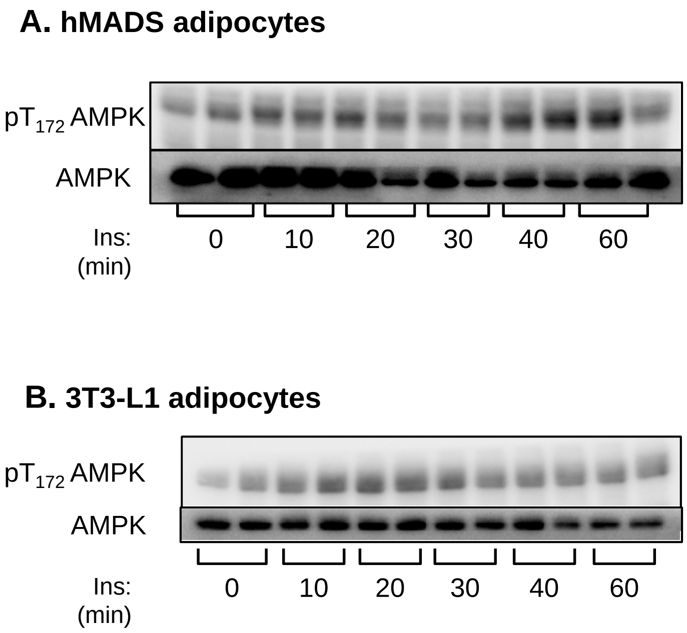


**Figure S1**

hMADS adipocytes (A) and 3T3-L1 adipocytes (B) were stimulated with insulin (100 nM) for the indicated period of times and analyzed by immunoblots with indicated antibodies.
