# Supplementary material for: Regulated in Development and DNA Damage Responses -1 (REDD1) Protein Contributes to Insulin Signaling Pathway in Adipocytes
Source: PLoS One. 2012 Dec 18;7(12):e52154. doi: 10.1371/journal.pone.0052154 (PMC3525563; doi:10.1371/journal.pone.0052154)
Supplement: Figure S2 — 3T3-L1 adipocytes were transfected with REDD1 siRNA (#1: s93077, #2: s93078, #3: 74747) as mentioned in materials and methods. 48 h after transfection, adipocytes were stimulated with insulin (10 nM) for 5 minutes. Proteins were analyzed by immunoblots using indicated antibodies. (PDF) [file pone.0052154.s002.pdf]

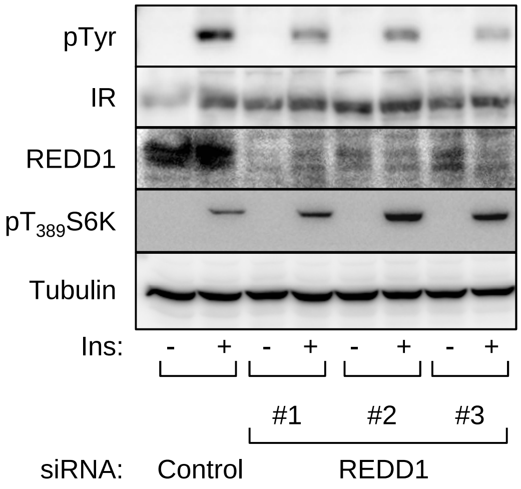

Figure S2

3T3-L1 adipocytes were transfected with REDD1 siRNA (#1: s93077, #2: s93078, #3: 74747) as mentioned in materials and methods. 48 h after transfection, adipocytes were stimulated with insulin (10 nM) for 5 minutes. Proteins were analyzed by immunoblots using indicated antibodies.
