# Supplementary material for: Regulated in Development and DNA Damage Responses -1 (REDD1) Protein Contributes to Insulin Signaling Pathway in Adipocytes
Source: PLoS One. 2012 Dec 18;7(12):e52154. doi: 10.1371/journal.pone.0052154 (PMC3525563; doi:10.1371/journal.pone.0052154)
Supplement: Figure S3 — 3T3-L1 adipocytes were transfected with REDD1 siRNA. Adipocytes were stimulated with insulin for 5 minutes. Proteins were analyzed by immunoblots using indicated antibodies. (PDF) [file pone.0052154.s003.pdf]

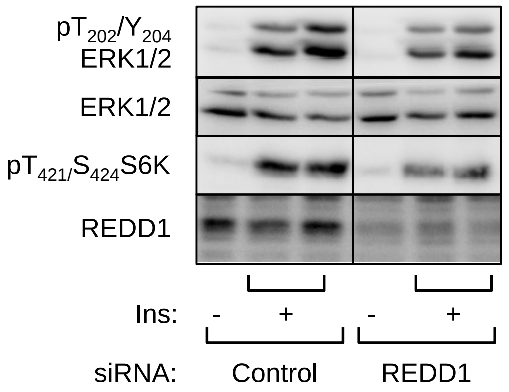

Figure S3

3T3-L1 adipocytes were transfected with REDD1 siRNA. Adipocytes were stimulated with insulin for 5 minutes. Proteins were analyzed by immunoblots using indicated antibodies.
